# MicroRNA-10b expression in breast cancer and its clinical association

**DOI:** 10.1371/journal.pone.0192509

**Published:** 2018-02-06

**Authors:** Jianhui Zhang, Jing Yang, Xin Zhang, Jia Xu, Yiyi Sun, Purong Zhang

**Affiliations:** 1 Sichuan Cancer Hospital & Institute, Sichuan Cancer Center, School of Medicine, University of Electronic Science and Technology of China, Chengdu, Sichuan, China; 2 Chengdu Medical College, Chengdu, Sichuan, China; University of South Alabama Mitchell Cancer Institute, UNITED STATES

## Abstract

MicroRNAs (miRNAs) are short non-coding RNA molecules that play a significant role in many types of cancers including breast cancer. In the current study, we evaluated the expression levels of microR-10b (miR-10b) in 115 breast cancer patients from Sichuan Cancer Center. Real time reverse transcription-PCR was used to assess miR-10b expression. Clinical data including disease stage, survival status, age, ER/PR/HER2 status, molecular subtypes, tumor size, lymph node status and Ki-67 expression levels were correlated with miR-10b expression levels. Our data showed that the miR-10b expression is correlated with disease stage, living status and tumor sizes. We also found that miR-10b expression levels are higher in the lymph node positive group and the Ki-67 higher scoring group (score > 20). No statistically significant differences were observed based on age or molecular sub-type grouping. In conclusion, miR-10b may be a biomarker for breast cancer and is a potential treatment target.

## Introduction

Breast cancer is the second most common cancer diagnosed worldwide with an estimated 249,260 new cases, and 40,890 deaths, estimated to occur in the United States during 2016 [1]. This makes breast cancer the second leading cancer type for estimated new cancer cases and deaths for 2016 [1]. In China, breast cancer is the second most common malignancy and the fourth leading cause of cancer-related death among females [1, 2]. In the past two decades, China has experienced an alarming increasing incidence of breast cancer [2]. Many breast tumors are known to respond to systemic treatments including surgery, chemotherapy or hormonal therapy. However, due to the heterogeneity of breast cancer, a significant number of patients will succumb to regional or distant metastases.

MicroRNAs or miRNAs are short non-coding RNA molecules (~22 nucleotides) that play important roles in gene regulation [3, 4]. Since the first microRNA lin-4 was identified in the nematode Caenorhabditis elegans [3], much research has been conducted in animals and humans. However, the complete function of human miRNAs is unclear compared to their roles in animal models [5].

In the past few decades, microRNA expression profiles in human breast cancers have led to conflicting reports [6–11]. Ma *et al*. [9] found higher miR-10b expression in patients with distant relapse, regional relapse and local recurrence while Gee *et al*. [11] reported opposite observations. Upregulation of miR-10b has been reported in glioblastoma, anaplastic astrocytomas, primary hepatocellular carcinomas and colon cancer [9, 12]. Recent studies show that breast cancer cell secreted miR-10b can be transported through exosomes and promote tumor development and progression [13]. In this study, we collected 125 pairs of primary breast cancers and matched non-cancerous breast tissue, as well as the clinical information, and correlated the relations between miR-10b expression levels to a variety of clinical outcomes.

## Materials and methods

### Patient cohort

The study was approved by the scientific ethic committees from Sichuan Cancer Hospital and Chengdu Medical College (IRB # HCHI-0158 and CMC-5020). Informed consent was obtained from all subjects. Information not related to current studies such as patient names, medical record numbers, national identity numbers, and contact information were de-identified in the studies. Tumor stage was assessed case by case by board certified pathologist following the national breast cancer stage guidelines (Table 1). 125 primary breast cancers tissue were obtained from patients from Sichuan Cancer Hospital from years 2000 to 2014. 115 of those samples demonstrated sufficient quality and quantity for miR-10b evaluation.

**Table 1:** Clinical and pathological data for the samples used in the study.

Clinical Feature	Case # (percentage)
**Stage** I	24 (21%)
II	42 (37%)
III	31 (27%)
IV	18 (16%)
**Survival Status**	
Alive	68 (59%)
Deceased	47 (41%)
**Age at diagnosis (Y)**	
≤50	42 (37%)
>50	73 (63%)
**ER Positive**	66 (57%)
**PR Positive**	52 (45%)
**Molecular Subtype**	
Luminal A	35 (30%)
Luminal B	28 (24%)
Basal-like	27 (23%)
HER2 Enriched	25 (22%)
**Tumor size (cm)**	
≤2	61 (53%)
>2	54 (47%)
**Lymph Node Status**	
Positive	44 (38%)
Negative	71 (62%)
**Ki-67 High Expression (≥20%)**	69 (60%)
**Recurrence**	44 (38%)

### Tumor selection and RNA extraction

In each case, the histopathological glass slides were microscopically reviewed by a pathologist to select the tumor block. Blocks with viable tumor tissue were chosen and ten sections of 10 μm thickness were cut from each selected block. One slide was chosen for H&E and four slides were chosen for immunohistochemical analysis for Ki-67, estrogen receptor (ER), progesterone receptor (PR), and HER2. An automated cellular imaging system was used to determine the Ki-67 percentage, ER, PR and HER2 scoring. For RNA extraction, a macro-dissection was performed on each sample and total RNA was extracted using miRNeasy FFPE kit (Qiagen, China) from formalin fixed paraffin embedded (FFPE) tumor tissue per manufacturer’s instructions. The quality of RNA was assessed by a Nanodrop spectrophotometer (Tecan, USA).

### Real time reverse transcription-polymerase chain reaction (RT-PCR)

MiR-10b expression levels were assessed using the qRT-PCR method. 5 ng of total RNA was used per sample to synthesize cDNA using the TaqMan MicroRNA Reverse Transcription kit (Applied Biosystems, China). Real time PCR was performed using the *mir*Vana qRT-PCR miRNA detection kit (Ambion^™^, ThermoFisher, China) according to the manufacturer’s instructions on the CFX96^™^ Real-Time PCR detection system (Bio-rad, China). The expression of U6 was used as the internal control and for RNA template normalization.

### Web-based analysis of genomic data sets

Mutation status, copy number, gene expression level and clinical information obtained from public repositories for miR-10b data were analyzed using the cBioPortal for Cancer Genomics (http://cbioportal.org) [14, 15]. Data was extracted and re-plotted.

### Statistical analysis

Data are presented as mean ± SEM. A Student’s T-test (for two groups) or one-way ANOVA (for three or more groups) was performed using SigmaPlot software (version 12.0, Systat) to determine significance between groups. Statistical significance was achieved when the p value is less than 0.05.

## Results

### MiR-10b expression levels correlate with malignant status in human breast cancer

**Fig 1:**
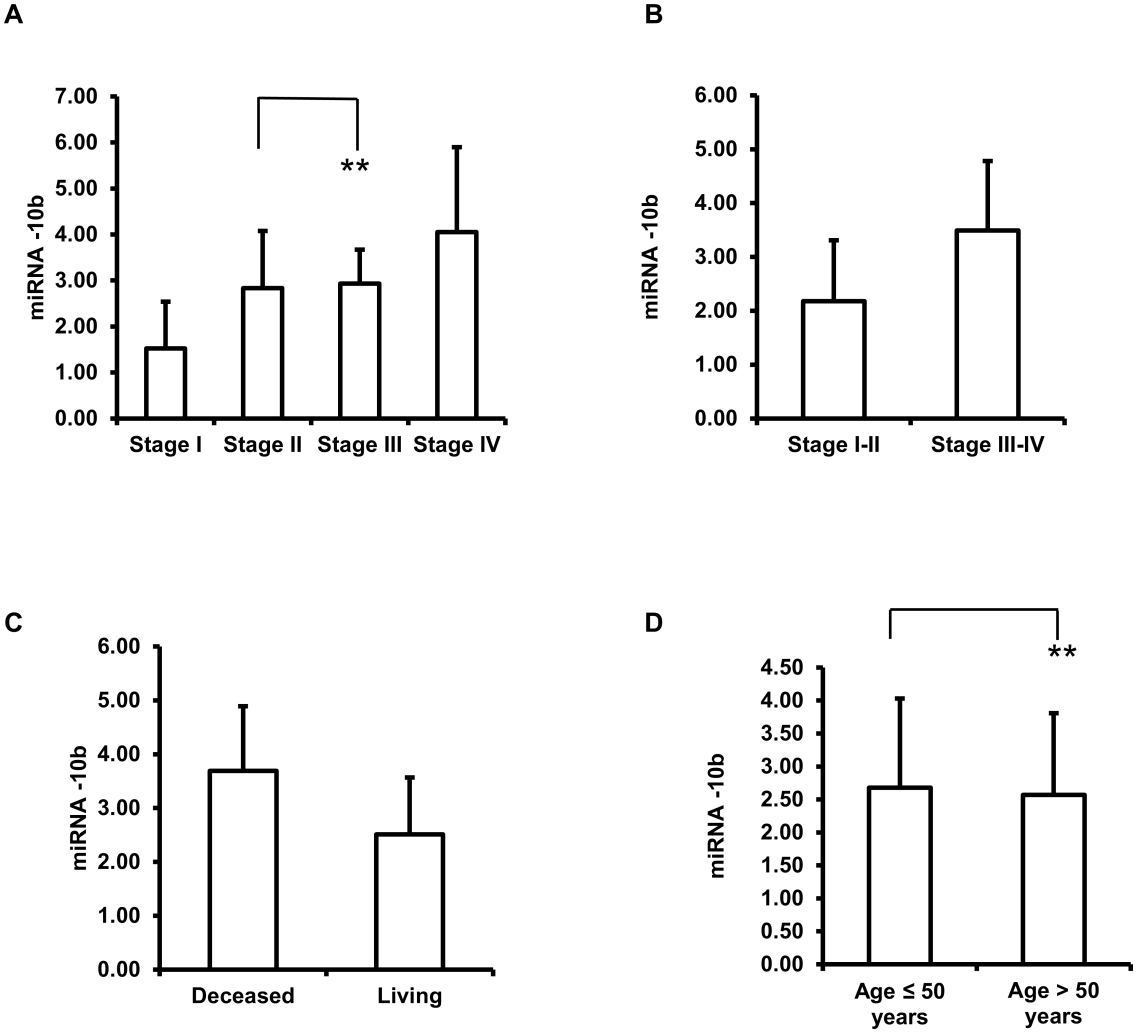
Expression of miR-10b in breast tumors. (A) miR-10b expression level fold changes based on stages. (B) miR-10b expression level fold changes based on stages. (C) miR-10b expression level fold changes based on living status. (D) miR-10b expression level fold changes based on ages. **p-value >0.05.

Expression levels of miR-10b were correlated with the breast cancer tumor stages. Stage I (n = 24, 1.52 ± 1.02 fold-change) miR-10b expression levels are significantly lower than the levels of stage II (n = 42, 2.84 ± 1.24 fold-change), Stage III (n = 31, 2.93 ± 0.74 fold-change) and Stage IV (n = 18, 4.05 ± 1.85 fold-change) (*p*-vale<0.01); Stage II miR-10b expression level is significantly lower than Stage IV (*p*-value = 0.002). There are no statistically significant changes observed between Stage II and III (*p*-value = 0.74). Stage III miR-10b expression level is significantly lower than Stage IV (*p*-value = 0.004) (Fig 1A). Significant expression level changes were also observed between stages I-II (n = 66, 2.18 ± 1.13 fold-change) and stages II-IV (n = 49, 3.49 ± 1.29 fold-change) (*p*-value<0.001) (Fig 1B).

### MiR-10b expression levels correlate with patient survival status but not to age

The miR-10b expression levels were assessed and correlated with patient survival status and ages (Fig 1C and 1D). Expression levels are significantly different between the living (n = 47, 2.51 ± 1.06 fold-change) and deceased (n = 68, 3.69 ± 1.20 fold-change) groups (*p*-value<0.001) (Fig 1C). Patients who are age 50 or less (n = 42, 2.68 ± 1.35 fold-change) have similar miR-10b expression levels as compared to patients who are older than 50 (n = 73, 2.57 ± 1.24 fold-change) (*p*-value = 0.62) (Fig 1D).

### No significant differences of miR-10b expression levels were observed based on HER2, ER, PR and molecular type grouping

**Fig 2:**
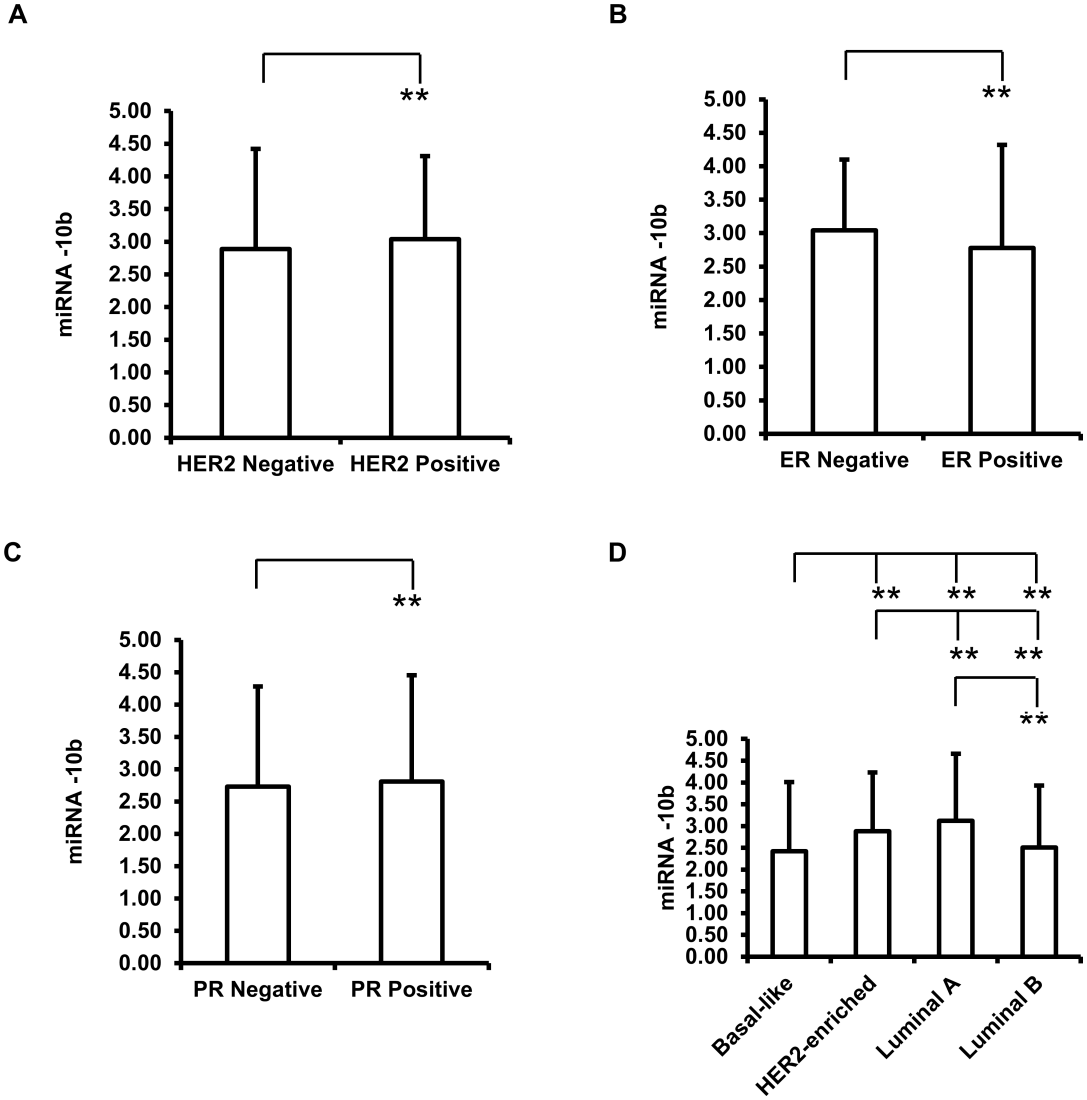
Expression of miR-10b in breast tumors. (A) miR-10b expression level fold changes based on HER2 status. (B) miR-10b expression level fold changes based on ER status. (C) miR-10b expression level fold changes based on PR status. (D) miR-10b expression level fold changes based on molecular subtypes. **p-value >0.05.

Patients who are HER2 negative (n = 90, 2.89 ± 1.53 fold-change) have similar miR-10b expression levels compared to patients (n = 25, 3.04 ± 1.27 fold-change) who are HER2 positive, *p*-value = 0.65 (Fig 2A). No significant difference in expression levels for miR-10b was detected for ER negative group (n = 49, 3.04 ± 1.06) and ER Positive group (n = 66, 2.78 ± 1.54) (*p*-value = 0.31) (Fig 2B). We also did not observe statistically significant different expression level changes between the PR Negative group (n = 63, 2.73 ± 1.55 fold-change) and PR Positive group (n = 52, 2.81 ± 1.65 fold-change) (*p*-value = 0.79) (Fig 2C). Grouping the expression data according to molecular subtype, we did not observe any significant differences in miR-10b expression levels between basal-like (n = 27, 2.42 ± 1.59 fold-change), HER2-enriched (n = 25, 2.88 ± 1.35 fold-change), Luminal A (n = 35, 3.12 ± 1.54 fold-change) and Luminal B (n = 28, 2.51 ± 1.42 fold-change) groups (Fig 2D).

### MiR-10b expression levels correlate with lymph nodes’ positivity/negativity, Ki-67 scores and tumor size

**Fig 3:**
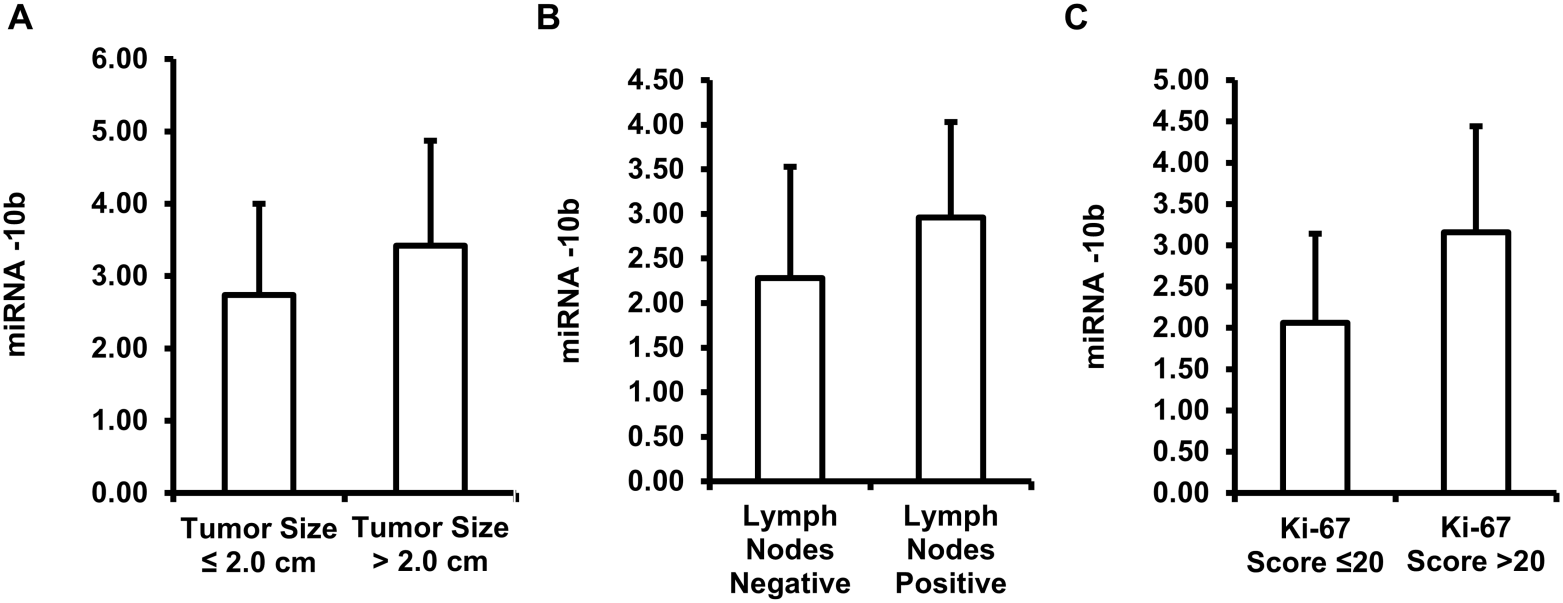
Expression of miR-10b in breast tumors. (A) miR-10b expression level fold changes based on tumor size. (B) miR-10b expression level fold changes based on lymph node status. (C) miR-10b expression level fold changes based on Ki-67 score.

We also investigated the relationship between expression levels of miR-10b and tumor size, lymph node numbers and Ki-67 scores. There is a significant difference between the expression levels of the patient group who has tumor size less than 2.0 cm (n = 61, 2.74 ± 1.26 fold-change) and the patient group with a tumor size larger than 2.0 cm (n = 54, 3.42 ± 1.45 fold-change) (*p*-value = 0.008) (Fig 3A). We found the expression levels of miR-10b are significantly lower in the lymph node negative groups (n = 71, 2.28 ± 1.25 fold-change) vs. the lymph nodes positive groups (n = 44, 2.96 ± 1.07 fold-change) (*p*-value = 0.003) (Fig 3B). The miR-10b expression levels are inversely correlated with the Ki-67 scores, the group with a Ki-67 score below or equal to 20 (n = 46, 2.06 ± 1.08 fold-change) has a lower expression level compared to the group with a Ki-67 score above 20 (n = 69, 3.06 ± 1.28 fold-change) (*p*-value<0.001) (Fig 3C).

### cBioPortal data analysis revealed the miR-10b amplification in different cancers

**Fig 4:**
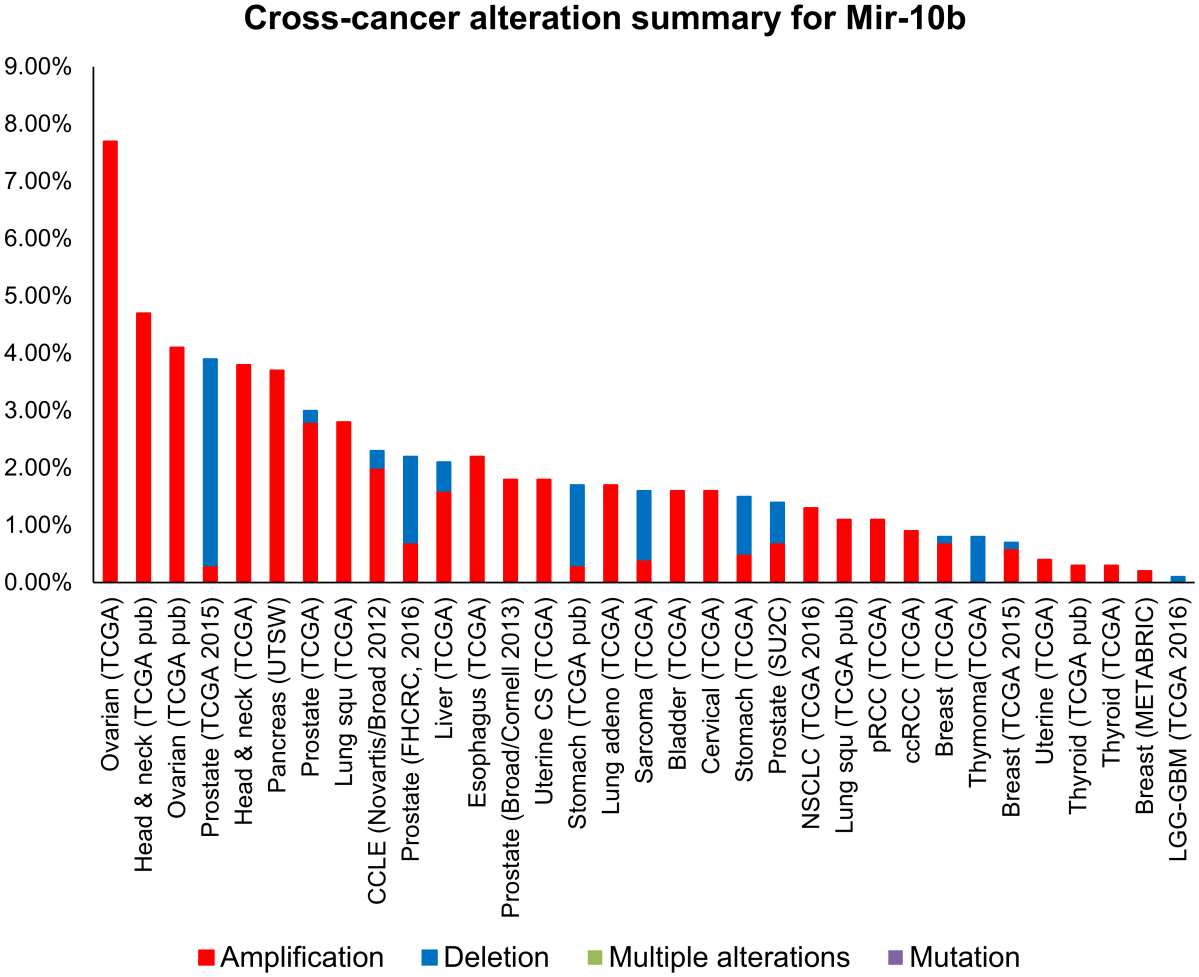
Cross-cancer alteration summary for miR-10b. A total of 147 studies were queried using the cBioPortal.

**Fig 5:**
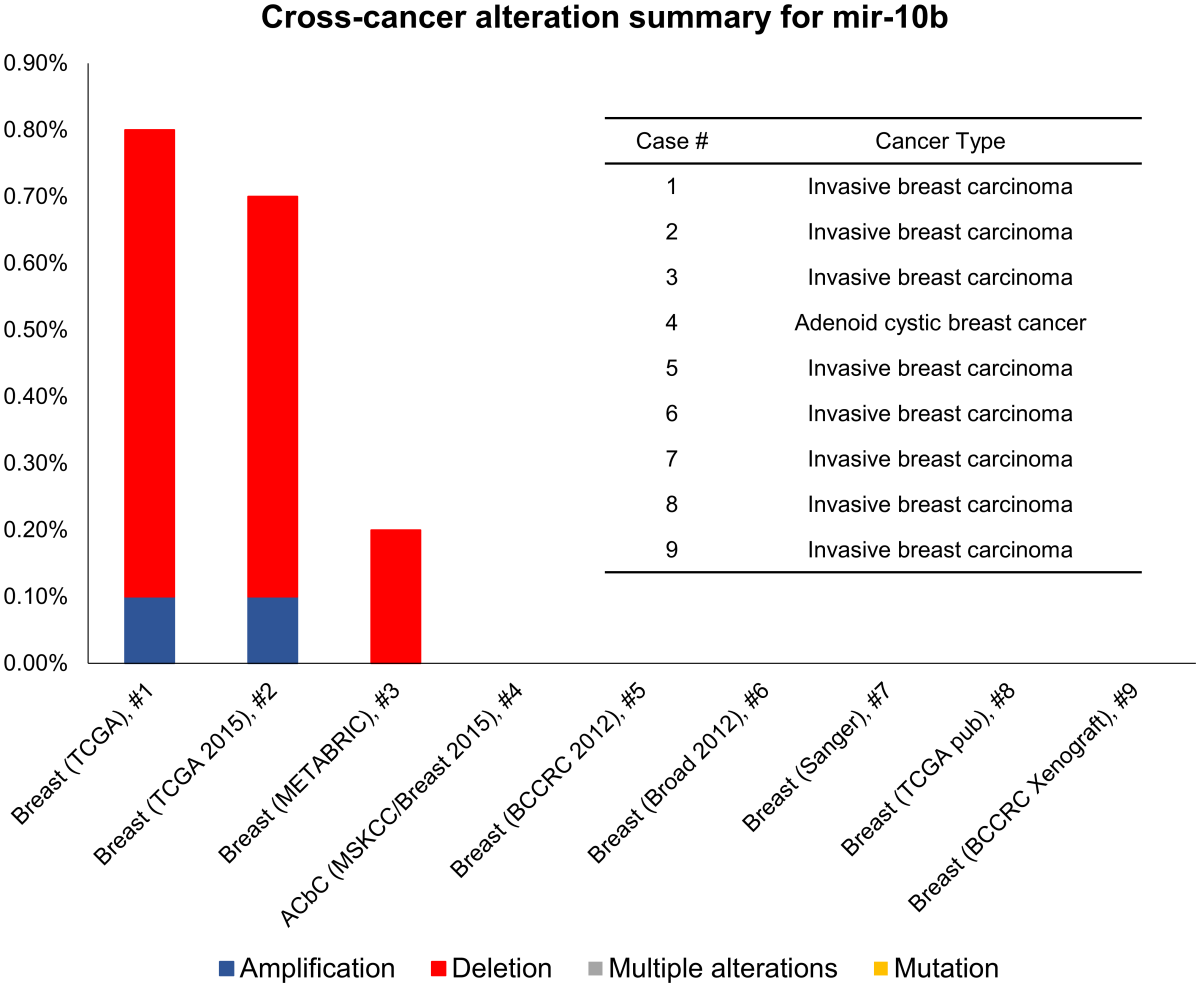
Cross-cancer alteration summary for miR-10b. A total of 9 breast invasive carcinoma studies were queried using the cBioPortal.

A total of 147 studies were queried using cBioPortal (S1 Table), 33 studies showed a minimum percentage of altered samples at 0.1% frequency and were re-plotted in Fig 4. The breast invasive carcinoma (TCGA, Provisional, n = 9) data showed a total of 0.7% cases has altered miR-10b expression, in which 0.6% showed amplification and 0.1% showed deletion (Fig 5).

## Discussion

In this work, we observed that miR-10b expression levels are inversely correlated with malignancy. There is a significant expression level difference between stage I and stage II-IV. However, there was no significant difference observed between stages II and III. We did not find any published data studying the correlations between the miR-10b expression levels and the disease stages.

We also observed a higher expression level of miR-10b in the deceased group versus the living group. However, the overall survival is dependent on multiple factors such as treatment received, other genetic status, other diseases status, economic and social status, etc. Based on age, patients who are 50 years or older have a lower miR-10b expression levels compared to the patients who are below 50 years old (not statistically significant). The lymph node positive group has higher miR-10b expression levels compared to the lymph node negative group. Based on Ki-67 score, the group with a score above 20 has higher miR-10b expression. Based on the comparison between disease stages, living status, lymph node status and the Ki-67 score and the miR-10b expression levels, it may indicate that the miR-10b expression level could be a prognostic biomarker.

Breast cancer is a heterogeneous disease and the PAM50 molecular subtypes of breast tumors, Luminal A, Luminal B, HER2-enriched, Basal-like, have distinct biological properties that can be used as markers for prognosis [16–20]. In our dataset, we did not find statistically significant changes in miR-10b expression levels among different molecular subtypes. The first couple of comprehensive reports on miRNA signatures for breast tumors reported that miR-125b, miR-145, miR-21 and miR-155 are the most dysregulated in these specimens. Some of these miRNAs were correlated with expression of hormone receptors, tumor stage, vascular invasion and proliferation but the correlation between expression and molecular subtypes were not mentioned [21, 22]. Earlier this year, a report on the correlation between miRNAs expression levels and molecular subtypes revealed that 17 miRNAs correlated to the subtypes, including hsa-miR-342-5p, hsa-miR-150, hsa-miR-155, hsa-miR-200c and hsa-miR-17 [23]. However, not all the miRNAs studies correlated with molecular subtypes [23].

Lim *et al*., identified miR-10b (UGUUUAAGCCAAGAUGUCCCAU) in 2003 [24]. Ma *et al*. showed that miR-10b is highly expressed in metastatic breast cancer in human cells and in mouse models [9]. Positive correlations between miR-10b expression levels and cell migration and invasion were also observed [9]. One year later, a brief communication associated with the findings was published in argument of the correlations between the miR-10b expression levels and the clinical progression. In their article, Gee *et al*. found no significant association between miR-10b expression levels and metastasis or prognosis in a total of 219 patients with primary breast cancer [11]. In response to this article, Ma *et al*. clarified that the correlation was between the miR-10b expression level and the metastatic behavior, not the prognostic property [25]. Later, Ma *et al*. showed that silencing of miR-10b inhibits metastasis in a xenograft mouse model [26]. Others have reported down-regulated miR-10b expression levels in breast cancer and suggested that restoration of miR-10b expression might have therapeutic promise [22, 27–30].

We did not observe a significant difference of miR-10b expression levels based on the molecular type grouping and the tumor size grouping.

The cross-cancer alteration summary for miR-10b based on the TCGA datasets showed that ovarian cancers have the highest miR-10b amplification rate. Breast cancers also have miR-10b amplification in three invasive breast carcinomas. Interestingly, there is another miR product, namely miR-10b*. Biagioni *et al*. indicated that the less common miR products, miR-10b*, expression is downregulated in breast cancer specimens (n = 56) versus the matched peritumoural samples [30].

## Conclusions

The miR-10b plays an important yet unclear role in human breast cancer and may be a prognostic biomarker. In addition, circulating miR-10b may also play an important role or can be served as a biomarker if we could correlate the clinical outcomes with the expression levels. We believe more functional and clinical studies are required to further explore the role of miR-10b in breast cancer tumorigenesis.

## Supporting information

S1 Table cBioPortal data analysis. (PDF)
