# Supplementary material for: MicroRNA-10b expression in breast cancer and its clinical association
Source: PLoS One. 2018 Feb 6;13(2):e0192509. doi: 10.1371/journal.pone.0192509 (PMC5800653; doi:10.1371/journal.pone.0192509)
Supplement: S1 Table — (PDF) [file pone.0192509.s001.pdf]

Table S1. CBioPortal data analysis.

Case #	STUDY_ABBREVIATION	STUDY_NAME	NUM_OF_CASES_ ALTERED	PERCENT_CASES_ ALTERED
1	Ovarian (TCGA)	Ovarian Serous Cystadenocarcinoma (TCGA, Provisional)	24	7.70%
2	Head & neck (TCGA pub)	Head and Neck Squamous Cell Carcinoma (TCGA, Nature 2015)	13	4.70%
3	Ovarian (TCGA pub)	Ovarian Serous Cystadenocarcinoma (TCGA, Nature 2011)	13	4.10%
4	Prostate (TCGA 2015)	Prostate Adenocarcinoma (TCGA, Cell 2015)	13	3.90%
5	Head & neck (TCGA)	Head and Neck Squamous Cell Carcinoma (TCGA, Provisional)	19	3.80%
6	Pancreas (UTSW)	Pancreatic Cancer (UTSW, Nat Commun 2015)	4	3.70%
7	Prostate (TCGA)	Prostate Adenocarcinoma (TCGA, Provisional)	15	3%
8	Lung squ (TCGA)	Lung Squamous Cell Carcinoma (TCGA, Provisional)	5	2.80%
9	CCLE (Novartis/Broad 2012)	Cancer Cell Line Encyclopedia (Novartis/Broad, Nature 2012)	21	2.40%
10	Prostate (FHCRC, 2016)	Prostate Adenocarcinoma (Fred Hutchinson CRC, Nat Med 2016)	3	2.20%
11	Liver (TCGA)	Liver Hepatocellular Carcinoma (TCGA, Provisional)	8	2.20%
12	Esophagus (TCGA)	Esophageal Carcinoma (TCGA, Provisional)	4	2.20%
13	Prostate (Broad/Cornell 2013)	Prostate Adenocarcinoma (Broad/Cornell, Cell 2013)	1	1.80%
14	Uterine CS (TCGA)	Uterine Carcinosarcoma (TCGA, Provisional)	1	1.80%
15	Stomach (TCGA pub)	Stomach Adenocarcinoma (TCGA, Nature 2014)	5	1.70%
16	Lung adeno (TCGA)	Lung Adenocarcinoma (TCGA, Provisional)	4	1.70%
17	Sarcoma (TCGA)	Sarcoma (TCGA, Provisional)	4	1.60%
18	Bladder (TCGA)	Bladder Urothelial Carcinoma (TCGA, Provisional)	2	1.60%
19	Cervical (TCGA)	Cervical Squamous Cell Carcinoma and Endocervical Adenocarcinoma (TCGA, Provisional)	3	1.60%
20	Stomach (TCGA)	Stomach Adenocarcinoma (TCGA, Provisional)	6	1.50%
21	Prostate (SU2C)	Metastatic Prostate Cancer, SU2C/PCF Dream Team (Robinson et al., Cell 2015)	2	1.30%
22	NSCLC (TCGA 2016)	Pan-Lung Cancer (TCGA, Nat Genet 2016)	15	1.30%
23	Lung squ (TCGA pub)	Lung Squamous Cell Carcinoma (TCGA, Nature 2012)	2	1.10%

Case #	STUDY_ABBREVIATION	STUDY_NAME	NUM_OF_CASES_ ALTERED	PERCENT_CASES_ ALTERED
24	pRCC (TCGA)	Kidney Renal Papillary Cell Carcinoma (TCGA, Provisional)	3	1.10%
25	ccRCC (TCGA)	Kidney Renal Clear Cell Carcinoma (TCGA, Provisional)	4	0.90%
26	Breast (TCGA)	Breast Invasive Carcinoma (TCGA, Provisional)	8	0.80%
27	Thymoma(TCGA)	Thymoma (TCGA, Provisional)	1	0.80%
28	Breast (TCGA 2015)	Breast Invasive Carcinoma (TCGA, Cell 2015)	6	0.70%
29	Uterine (TCGA)	Uterine Corpus Endometrial Carcinoma (TCGA, Provisional)	1	0.40%
30	Thyroid (TCGA pub)	Papillary Thyroid Carcinoma (TCGA, Cell 2014)	1	0.30%
31	Thyroid (TCGA)	Thyroid Carcinoma (TCGA, Provisional)	1	0.30%
32	Breast (METABRIC)	Breast Cancer (METABRIC, Nature 2012 & Nat Commun 2016)	5	0.20%
33	LGG-GBM (TCGA 2016)	Merged Cohort of LGG and GBM (TCGA, Cell 2016)	1	0.10%
34	Lung adeno (TCGA pub)	Lung Adenocarcinoma (TCGA, Nature 2014)	0	0%
35	Colorectal (Genentech)	Colorectal Adenocarcinoma (Genentech, Nature 2012)	0	0%
36	Colorectal (TCGA pub)	Colorectal Adenocarcinoma (TCGA, Nature 2012)	0	0%
37	Colorectal (TCGA)	Colorectal Adenocarcinoma (TCGA, Provisional)	0	0%
38	Colorectal (MSKCC)	Colorectal Adenocarcinoma Triplets (MSKCC, Genome Biol 2014)	0	0%
39	CTCL (Columbia 2015)	Cutaneous T Cell Lymphoma (Columbia U, Nat Genet 2015)	0	0%
40	CSCC (DFCI 2015)	Cutaneous squamous cell carcinoma (DFCI, Clin Cancer Res 2015)	0	0%
41	DESM (Broad 2015)	Desmoplastic Melanoma (Broad Institute, Nat Genet 2015)	0	0%
42	DLBCL (Broad 2012)	Diffuse Large B-Cell Lymphoma (Broad, PNAS 2012)	0	0%
43	Esophagus (Broad)	Esophageal Adenocarcinoma (Broad, Nat Genet 2013)	0	0%
44	ACbC (MSKCC/Breast 2015)	Adenoid Cystic Carcinoma of the Breast (MSKCC, J Pathol. 2015)	0	0%
45	Esophagus sq (ICGC)	Esophageal Squamous Cell Carcinoma (ICGC, Nature 2014)	0	0%
46	ESCC (UCLA 2014)	Esophageal Squamous Cell Carcinoma (UCLA, Nat Genet 2014)	0	0%
47	Ewing Sarcoma (Institut Curie)	Ewing Sarcoma (Institut Cuire, Cancer Discov 2014)	0	0%

Case #	STUDY_ABBREVIATION	STUDY_NAME	NUM_OF_CASES_ ALTERED	PERCENT_CASES_ ALTERED
48	GBC (Shanghai)	Gallbladder Carcinoma (Shanghai, Nat Genet 2014)	0	0%
49	Stomach (TMUCIH 2015)	Gastric Adenocarcinoma (TMUCIH, PNAS 2015)	0	0%
50	GBM (TCGA 2013)	Glioblastoma (TCGA, Cell 2013)	0	0%
51	GBM (TCGA 2008)	Glioblastoma (TCGA, Nature 2008)	0	0%
52	GBM (TCGA)	Glioblastoma Multiforme (TCGA, Provisional)	0	0%
53	Head & neck (Broad)	Head and Neck Squamous Cell Carcinoma (Broad, Science 2011)	0	0%
54	Head & neck (JHU)	Head and Neck Squamous Cell Carcinoma (Johns Hopkins, Science 2011)	0	0%
55	ACC (TCGA)	Adrenocortical Carcinoma (TCGA, Provisional)	0	0%
56	Bladder (MSKCC 2014)	Bladder Cancer (MSKCC, Eur Urol 2014)	0	0%
57	Liad (Inserm 2014)	Hepatocellular Adenoma (Inserm, Cancer Cell 2014)	0	0%
58	ALL (St. Jude)	Hypodiploid Acute Lymphoid Leukemia (St Jude, Nat Genet 2013)	0	0%
59	ALL (St. Jude)	Infant MLL-Rearranged Acute Lymphoblastic Leukemia (St Jude, Nat Genet 2015)	0	0%
60	Panet (Shanghai 2013)	Insulinoma (Shanghai, Nat Commun 2013)	0	0%
61	Cholangiocarcinoma (JHU)	Intrahepatic Cholangiocarcinoma (Johns Hopkins University, Nat Genet 2013)	0	0%
62	chRCC (TCGA)	Kidney Chromophobe (TCGA, Cancer Cell 2014)	0	0%
63	chRCC (TCGA)	Kidney Chromophobe (TCGA, Provisional)	0	0%
64	ccRCC (BGI 2012)	Kidney Renal Clear Cell Carcinoma (BGI, Nat Genet 2012)	0	0%
65	ccRCC (TCGA pub)	Kidney Renal Clear Cell Carcinoma (TCGA, Nature 2013)	0	0%
66	Bladder (MSKCC 2012)	Bladder Cancer (MSKCC, JCO 2013)	0	0%
67	Bladder PV (MSKCC)	Bladder Cancer, Plasmacytoid Variant (MSKCC, Nat Genet 2016)	0	0%
68	Liver (AMC)	Liver Hepatocellular Carcinoma (AMC, Hepatology 2014)	0	0%
69	Liver (RIKEN)	Liver Hepatocellular Carcinoma (RIKEN, Nat Genet 2012)	0	0%
70	Bladder (BGI 2013)	Bladder Urothelial Carcinoma (BGI, Nat Genet 2013)	0	0%
71	Glioma (UCSF)	Low-Grade Gliomas (UCSF, Science 2014)	0	0%
72	Lung adeno (Broad)	Lung Adenocarcinoma (Broad, Cell 2012)	0	0%

Case #	STUDY_ABBREVIATION	STUDY_NAME	NUM_OF_CASES_ ALTERED	PERCENT_CASES_ ALTERED
73	Lung adeno (MSKCC)	Lung Adenocarcinoma (MSKCC 2015)	0	0%
74	AML (TCGA pub)	Acute Myeloid Leukemia (TCGA, NEJM 2013)	0	0%
75	Bladder (DFCI/MSKCC 2014)	Bladder Urothelial Carcinoma (Dana Farber & MSKCC, Cancer Discov 2014)	0	0%
76	Lung adeno (TSP)	Lung Adenocarcinoma (TSP, Nature 2008)	0	0%
77	Bladder (TCGA 2014)	Bladder Urothelial Carcinoma (TCGA, Nature 2014)	0	0%
78	PAAC (JHU)	Acinar Cell Carcinoma of the Pancreas (Johns Hopkins, J Pathol 2014)	0	0%
79	DLBC (TCGA)	Lymphoid Neoplasm Diffuse Large B-cell Lymphoma (TCGA, Provisional)	0	0%
80	MPNST (MSKCC)	Malignant Peripheral Nerve Sheath Tumor (MSKCC, Nat Genet 2014)	0	0%
81	PLMESO (NYU 2015)	Malignant Pleural Mesothelioma (NYU, Cancer Res 2015)	0	0%
82	MCL (IDIBIPS 2013)	Mantle Cell Lymphoma (IDIBIPS, PNAS 2013)	0	0%
83	MBL (Broad)	Medulloblastoma (Broad, Nature 2012)	0	0%
84	MBL (ICGC)	Medulloblastoma (ICGC, Nature 2012)	0	0%
85	MBL (PCGP)	Medulloblastoma (PCGP, Nature 2012)	0	0%
86	Melanoma (Broad/DFCI)	Melanoma (Broad/Dana Farber, Nature 2012)	0	0%
87	Glioma (TCGA)	Brain Lower Grade Glioma (TCGA, Provisional)	0	0%
88	Mesothelioma (TCGA)	Mesothelioma (TCGA, Provisional)	0	0%
89	AML (TCGA)	Acute Myeloid Leukemia (TCGA, Provisional)	0	0%
90	MM (Broad)	Multiple Myeloma (Broad, Cancer Cell 2014)	0	0%
91	ccRCC (IRC)	Multiregion Sequencing of Clear Cell Renal Cell Carcinoma (IRC, Nat Genet 2014).	0	0%
92	MDS (Tokyo)	Myelodysplasia (Tokyo, Nature 2011)	0	0%
93	NCI-60	NCI-60 Cell Lines (NCI, Cancer Res. 2012)	0	0%
94	NPC (Singapore)	Nasopharyngeal Carcinoma (Singapore, Nat Genet 2014)	0	0%
95	NBL (AMC)	Neuroblastoma (AMC Amsterdam, Nature 2012)	0	0%
96	NBL (Cologne 2015)	Neuroblastoma (Broad, Nat Genet 2013)	0	0%
97	NEPC (Trento/Cornell/Broad 2016)	Neuroendocrine Prostate Cancer (Trento/Cornell/Broad 2016)	0	0%

Case #	STUDY_ABBREVIATION	STUDY_NAME	NUM_OF_CASES_ALTERED	PERCENT_CASES_ALTERED
98	Head & neck (MDA)	Oral Squamous Cell Carcinoma (MD Anderson, Cancer Discov 2013)	0	0%
99	Breast (BCCRC 2012)	Breast Invasive Carcinoma (British Columbia, Nature 2012)	0	0%
100	Breast (Broad 2012)	Breast Invasive Carcinoma (Broad, Nature 2012)	0	0%
101	Breast (Sanger)	Breast Invasive Carcinoma (Sanger, Nature 2012)	0	0%
102	Pancreas (ICGC)	Pancreatic Adenocarcinoma (ICGC, Nature 2012)	0	0%
103	Pancreas (QCMG 2016)	Pancreatic Adenocarcinoma (QCMG, Nature 2016)	0	0%
104	Pancreas (TCGA)	Pancreatic Adenocarcinoma (TCGA, Provisional)	0	0%
105	ACyC (FMI 2014)	Adenoid Cystic Carcinoma (FMI, Am J Surg Pathl. 2014)	0	0%
106	PANET(Johns Hopkins 2011)	Pancreatic Neuroendocrine Tumors (Johns Hopkins University, Science 2011)	0	0%
107	Breast (TCGA pub)	Breast Invasive Carcinoma (TCGA, Nature 2012)	0	0%
108	Ewing Sarcoma (DFCI)	Pediatric Ewing Sarcoma (DFCI, Cancer Discov 2014)	0	0%
109	PCPG (TCGA)	Pheochromocytoma and Paraganglioma (TCGA, Provisional)	0	0%
110	Thyroid (MSKCC 2016)	Poorly-Differentiated and Anaplastic Thyroid Cancers (MSKCC, JCI 2016)	0	0%
111	PCNSL (Mayo Clinic)	Primary Central Nervous System Lymphoma (Mayo Clinic, Clin Cancer Res 2015)	0	0%
112	ACyC (MDA 2015)	Adenoid Cystic Carcinoma (MDA, Clin Cancer Res 2015)	0	0%
113	Prostate (Broad/Cornell 2012)	Prostate Adenocarcinoma (Broad/Cornell, Nat Genet 2012)	0	0%
114	Breast (BCCRC Xenograft)	Breast cancer patient xenografts (British Columbia, Nature 2014)	0	0%
115	Prostate (MSKCC 2010)	Prostate Adenocarcinoma (MSKCC, Cancer Cell 2010)	0	0%
116	ACyC (MSKCC, 2013)	Adenoid Cystic Carcinoma (MSKCC, Nat Genet 2013)	0	0%
117	ACyC (Sanger 2013)	Adenoid Cystic Carcinoma (Sanger/MDA, JCI 2013)	0	0%
118	Prostate (MSKCC 2014)	Prostate Adenocarcinoma CNA study (MSKCC, PNAS 2014)	0	0%
119	Prostate Organoids	Prostate Adenocarcinoma Organoids (MSKCC, Cell 2014)	0	0%
120	Prostate (MICH)	Prostate Adenocarcinoma, Metastatic (Michigan, Nature 2012)	0	0%

Case #	STUDY_ABBREVIATION	STUDY_NAME	NUM_OF_CASES_ ALTERED	PERCENT_CASES_ ALTERED
121	hnc_mskcc_2016	Recurrent and Metastatic Head & Neck Cancer (JAMA Oncology, 2016)	0	0%
122	nccRCC (Genentech 2014)	Renal Non-Clear Cell Carcinoma (Genentech, Nat Genet 2014)	0	0%
123	RMS (NIH)	Rhabdomyosarcoma (NIH, Cancer Discov 2014)	0	0%
124	Sarcoma (MSKCC)	Sarcoma (MSKCC/Broad, Nat Genet 2010)	0	0%
125	Cholangiocarcinoma (NCCS)	Cholangiocarcinoma (National Cancer Centre of Singapore, Nat Genet 2013)	0	0%
126	Melanoma (Broad)	Skin Cutaneous Melanoma (Broad, Cell 2012)	0	0%
127	Melanoma (TCGA)	Skin Cutaneous Melanoma (TCGA, Provisional)	0	0%
128	Melanoma (Yale)	Skin Cutaneous Melanoma (Yale, Nat Genet 2012)	0	0%
129	Ovary SC (MSKCC)	Small Cell Carcinoma of the Ovary (MSKCC, Nat Genet 2014)	0	0%
130	Small Cell Lung (CLCGP)	Small Cell Lung Cancer (CLCGP, Nat Genet 2012)	0	0%
131	Small Cell Lung (JHU)	Small Cell Lung Cancer (Johns Hopkins, Nat Genet 2012)	0	0%
132	Small Cell Lung (UCOLOGNE)	Small Cell Lung Cancer (U Cologne, Nature 2015)	0	0%
133	Stomach (Pfizer UHK)	Stomach Adenocarcinoma (Pfizer and UHK, Nat Genet 2014)	0	0%
134	Cholangiocarcinoma (NUS)	Cholangiocarcinoma (National University of Singapore, Nat Genet 2012)	0	0%
135	Cholangiocarcinoma (TCGA)	Cholangiocarcinoma (TCGA, Provisional)	0	0%
136	Stomach (UTokyo)	Stomach Adenocarcinoma (U Tokyo, Nat Genet 2014)	0	0%
137	Stomach (UHK)	Stomach Adenocarcinoma (UHK, Nat Genet 2011)	0	0%
138	Testicular germ cell (TCGA)	Testicular Germ Cell Cancer (TCGA, Provisional)	0	0%
139	TET (NCI)	Thymic Epithelial Tumors (NCI, Nat Genet 2014)	0	0%
140	CLL (Broad 2013)	Chronic Lymphocytic Leukemia (Broad, Cell 2013)	0	0%
141	CLL(IUOPA 2015)	Chronic Lymphocytic Leukemia (IUOPA, Nature 2015)	0	0%
142	Renal unclass (MSKCC)	Unclassified Renal Cell Carcinoma (MSKCC 2016)	0	0%
143	ucs (Johns Hopkins 2014)	Uterine Carcinosarcoma (Johns Hopkins University, Nat Commun 2014)	0	0%

Case #	STUDY_ABBREVIATION	STUDY_NAME	NUM_OF_CASES_ ALTERED	PERCENT_CASES_ ALTERED
144	ccRCC (U Tokyo)	Clear Cell Renal Cell Carcinoma (U Tokyo, Nat Genet 2013)	0	0%
145	Uterine (TCGA pub)	Uterine Corpus Endometrial Carcinoma (TCGA, Nature 2013)	0	0%
146	Colorectal (DFCI 2016)	Colorectal Adenocarcinoma (DFCI, Cell Reports 2016)	0	0%
147	Uveal melanoma (TCGA)	Uveal Melanoma (TCGA, Provisional)	0	0%
